# Impacts of Climate Change on the Distribution of Suitable Habitat for Invasive *Coreopsis* Species in China

**DOI:** 10.1002/ece3.73073

**Published:** 2026-02-10

**Authors:** Jinglin Jia, Junwei Ye, Jianjun Zeng

**Affiliations:** ^1^ School of Life Sciences, Key Laboratory of Jiangxi Province for Biological Invasion and Biosecurity Jinggangshan University Ji'an China

**Keywords:** climate change, *Coreopsis* genus, MaxEnt model, niche overlap, suitable habitat

## Abstract

Climate change poses a serious threat to global species distributions and has significantly altered the distribution patterns of invasive species. *Coreopsis* spp. are widely distributed invasive plants with strong adaptability and reproductive capacity, whose invasion has become a major ecological concern in China. Using three climate change scenarios (SSP‐126, SSP‐245, SSP‐585), combined with the Maximum Entropy (MaxEnt) model and Geographic Information System (ArcGIS), this study delineated the potential distribution areas and distribution centroids of invasive *Coreopsis* plants in China. The results indicated that temperature (especially isothermality BIO3 and mean temperature of the warmest quarter BIO10) and moisture are the primary climatic factors influencing the distribution of *Coreopsis* spp., while human activity (HA) also plays a key role in shaping their distribution. *Coreopsis drummondii* exhibited the largest suitable habitat area (4.138 × 10^6^ km^2^), whereas *Coreopsis verticillata* had the smallest (9.53 × 10^5^ km^2^). Under current climatic conditions, the six *Coreopsis* species are mainly distributed in southern China. In future climate scenarios, their distributions are projected to shift northward and toward plateau regions. Moreover, high niche and range overlap was observed among *Coreopsis grandiflora* , *Coreopsis lanceolata* , and *Coreopsis tinctoria* , suggesting potential intensified interspecific competition. This study systematically reveals the invasion potential and spatial dynamics of *Coreopsis* spp. under climate change, providing a scientific basis for early warning, regional management, and ecological control. It also offers perspectives for future research on the interaction mechanisms between invasive and native species.

## Graphical abstract

Here's a concise 3‐sentence summary: This study uses climate models and mapping to predict how climate change will shift the suitable habitats for six invasive “Coreopsis” plant species in China, currently concentrated in the south. Key findings include a projected northward and plateau migration, differing environmental drivers (temperature, humidity, or human activity) for each species, and significant overlap suggesting competition between some species. The research provides crucial scientific evidence for assessing and managing the ecological risks posed by these invasions.

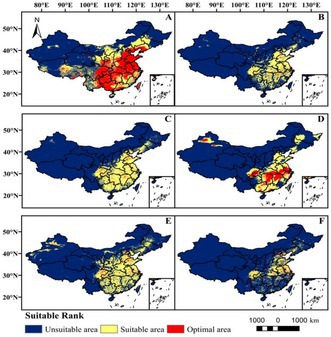

## 1 Introduction

Global climate change is significantly altering the geographical distribution patterns of plant species, particularly the spread rate and range of invasive species (Diagne et al. 2021; Osunkoya et al. 2025). Climate factors directly or indirectly regulate the invasion process and establishment success of species by influencing their niche characteristics (Chen et al. 2024; Qi et al. 2023). In recent years, with the continuous increase in greenhouse gas emissions, rising surface temperatures and changes in precipitation patterns have driven many species to migrate toward higher latitudes and altitudes at a pace far exceeding historical norms (Feeley and Freeman 2023).

The colonization and spread of invasive plants in new environments often result from the synergistic effects of biotic and environmental factors. Among these, climate change not only alters the physiologically suitable ranges of species but may also create competitive advantages for invasive species through indirect pathways such as modifying community structure and resource availability (Ehrlén and Morris 2015; Pecl et al. 2017). Therefore, elucidating the mechanisms by which climate change affects the distribution of invasive plants is of great significance for building early warning systems for biological invasions and formulating adaptive management strategies.

*Coreopsis* species, native to the Americas and Africa, are characterized by high reproductive capacity and stress tolerance (Chinese Academy Of Sciences 1979). They were introduced to China as ornamental plants and have been widely cultivated (Zhang 2013). However, their strong adaptability and dispersal ability have led to their escape and establishment as invasive populations in many regions, posing threats to local biodiversity and ecosystem functioning (Arifin and Okamoto 2023; Eunhee and Deokjoo 2024). Currently, systematic research on the potential distribution patterns of *Coreopsis* species in China and their responses to climate change is still lacking.

Based on this, this study selected six *Coreopsis* species that have established populations in China ( *C. drummondii* , *C. grandiflora* , *C. lanceolata* , *C. major* , *C. tinctoria* , *C. verticillata* ) (Chinese Academy Of Sciences 1979). The aims are to: (1) identify the key environmental factors influencing their distribution; (2) predict changes in their potential suitable habitats under future climate change; and (3) assess the degree of niche overlap among species to explore the possibility of interspecific competition. The findings will provide a theoretical basis for invasion risk assessment, regional management, and ecological control of *Coreopsis* species.

## 2 Materials and Methods

### 2.1 Species Distribution Data

**FIGURE 1:**
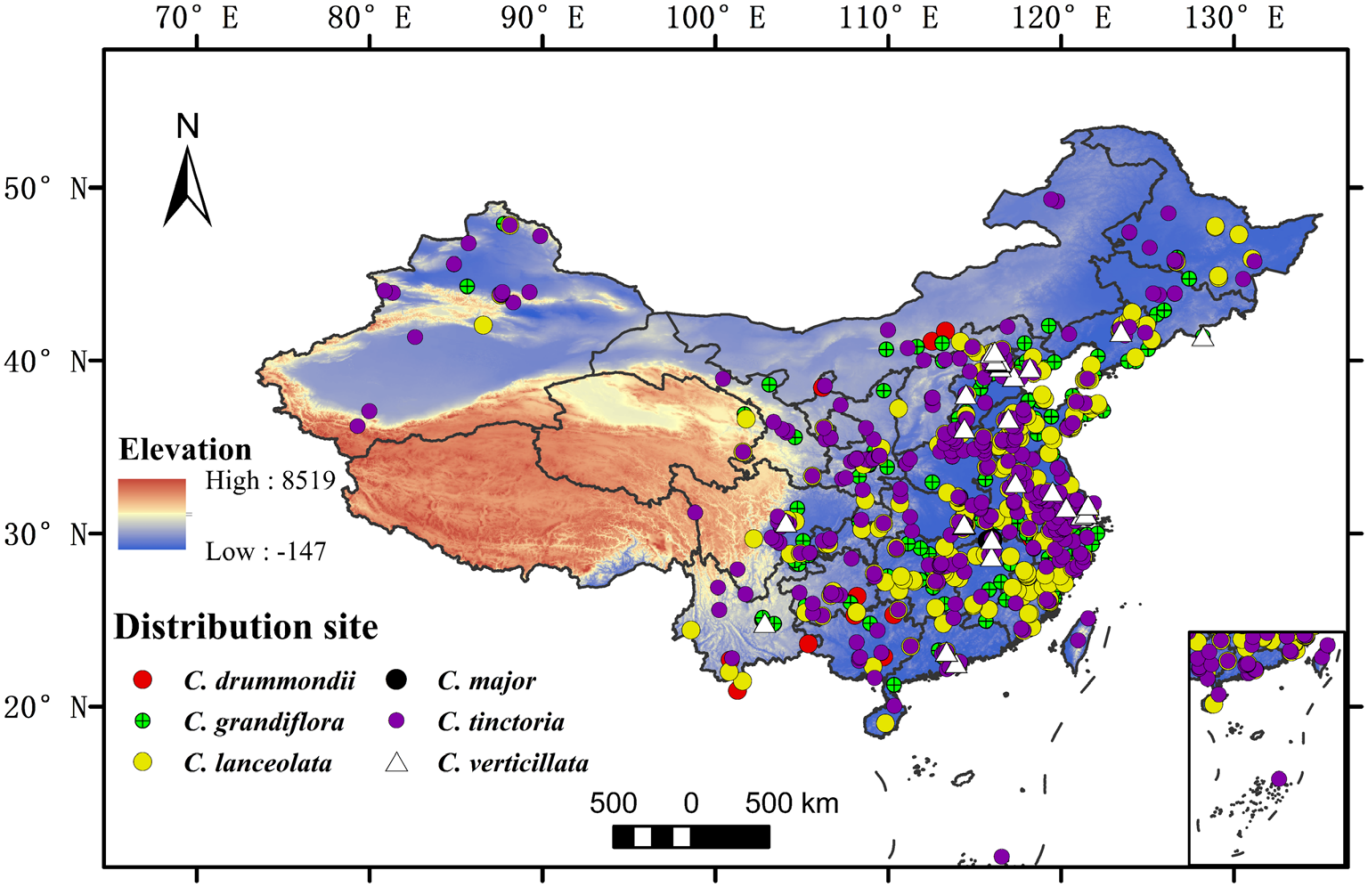
The distribution records of 6 species *Coreopsis* in China (12 records of *C. drummondii* , 249 records of *C. grandiflora* , 211 records of *C. lanceolata* , 5 records of *C. major* , 249 records of *C. tinctoria* , 24 records of *C. verticillata* ).

The distribution records of six species of the *Coreopsis* genus in China are shown in Figure 1. This study obtained 1085 distribution points through field surveys and online data retrieval from the Global Biodiversity Information Facility (GBIF, https://www.gbif.org/, accessed on January 16, 2025), the Chinese Virtual Herbarium Database (CVH, https://www.cvh.ac.cn/, accessed on January 16, 2025), and the National Specimen Information Infrastructure Database (NSII, http://nsii.org.cn/2017/home.php, accessed on January 16, 2025)., The records with missing or invalid coordinates, duplicates, and redundant data were excluded. ENMTools were used to remove duplicate or similar distribution points within a 1 km radius, and records without coordinates (Zhang et al. 2018). This resulted in 750 valid distribution points.

### 2.2 Environmental Variable Collection

Environmental variables were obtained from the WorldClim database (https://www.worldclim.org/, accessed on April 23, 2023). This included 19 climate factors related to current and future climate scenarios (average values for 2041–2060 and 2081–2100), with data provided by the Coupled Model Intercomparison Project Phase 6 (CMIP6) at a spatial resolution of 2.5 arc‐minutes (approximately 5 km^2^). Future climate data was selected from the medium‐resolution climate system model (BCC‐CSM2‐MR) of the Beijing Climate Center. This system provides the most suitable climate data for climate change studies in China. Three Shared Socioeconomic Pathways (SSPs) were set for future climate change scenarios: SSP126 (temperature rise of 1.0°C–2.4°C by 2100), SSP245 (temperature rise of 3.3°C–7.6°C by 2100), and SSP585 (temperature rise of 1.7°C–3.8°C by 2100) (Zhang et al. 2018). Different greenhouse gas emission scenarios lead to varying temperature increases under the three future climate scenarios (Meinshausen et al. 2020). Elevation, slope, and aspect data were obtained from a 25 m‐resolution digital elevation model (DEM), sourced from the Institute of Computing Technology, Chinese Academy of Sciences and the International Science Data Centre (http://www.gscloud.cn/, accessed on April 23, 2023). Human activity (HA) data, represented by the Global Human Influence Index, were obtained on April 23, 2023, from the Socioeconomic Data and Applications Center (SEDAC: http://sedac.ciesin.columbia.edu/wildareas/).

### 2.3 Niche Modeling

This study used the MaxEnt 3.4.1 model to predict the potential distribution patterns of six species of *Coreopsis* under 23 environmental variables. The model used the Jackknife method to quantify the contribution of environmental factors, using 75% of the distribution data for training and 25% for testing, with other parameters set to their default values (Zhong et al. 2023). Model accuracy was assessed using the receiver operating characteristic (ROC) curve, with the area under the curve (AUC) as the evaluation metric. The grading standards were as follows: 0.5–0.6 (poor prediction), 0.6–0.7 (fair), 0.7–0.8 (good), 0.8–0.9 (very good), and 0.9–1.0 (excellent) (Wan et al. 2019). The results indicated that the models developed in this study had AUC values greater than 0.9, demonstrating a high level of reliability in the predictions.

### 2.4 Habitat Suitability Delineation

The MaxEnt model results were imported into ArcMap software (Version 10.8), and the “Reclassification” tool was used to delineate suitable areas. The maximum training sensitivity plus specificity (MTSS) was selected as the threshold for defining suitable and unsuitable regions, as it represents the most conservative value, minimizing omission errors (Abdelaal et al. 2019; Ngarega et al. 2022). Based on MTSS data, the potential distribution area was classified into three categories: unsuitable areas (<MTSS), suitable areas (MTSS‐0.60), and highly suitable areas (> 0.60). This approach is consistent with previous studies (Abdelaal et al. 2019; Chang et al. 2025; Choi and Lee 2022). Subsequently, the pixel points of suitable areas were converted into regions using ArcGIS 10.8.

### 2.5 Centroid Distribution Analysis

The ArcGIS software (SDMtoolbox v2.4) was used to calculate the proportion and area of potential distribution zones for six species of the genus *Coreopsis* under current and future climate change scenarios. Additionally, the contraction, expansion, and stability of their suitable habitats, along with centroid shifts, were analyzed (Zhang et al. 2018), and maps for potential habitat changes and centroid migration routes for the six species were generated.

### 2.6 Niche and Range Overlap Analysis

To quantify the similarity of suitable habitats for six species of *Coreopsis* in the study area, ENMTools v1.3 software was used to calculate the niche overlap index D (Schoener's D) (Schoener 1968) and I (Hellinger‐based I) (Warren et al. 2008). These values ranged from 0 to 1, with values closer to 1 indicating greater similarity in the species' niche distributions.

## 3 Results

### 3.1 Model Performance and Key Environmental Variables

**FIGURE 2:**
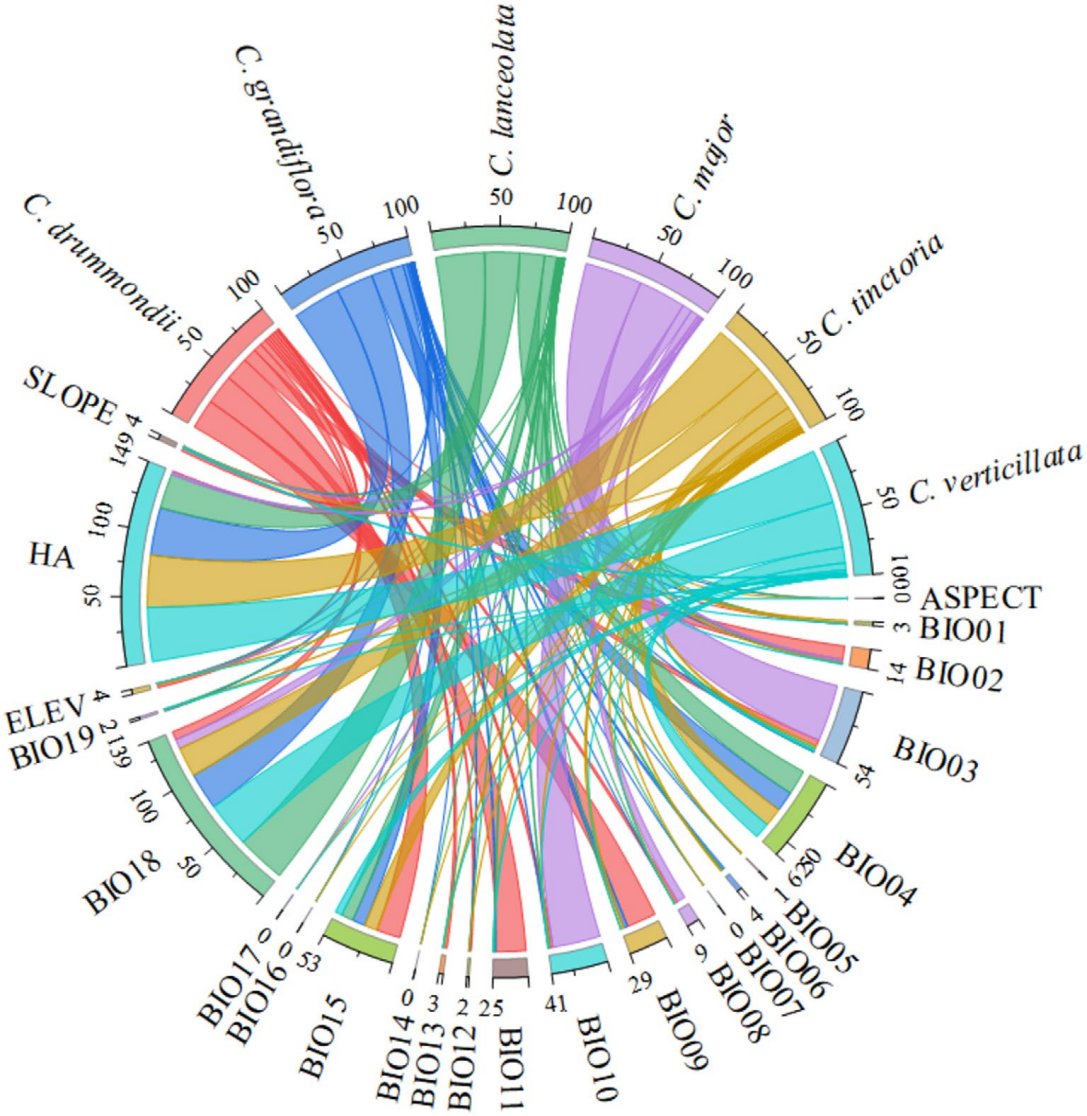
Chord plot of the cumulative contribution of major climatic factors of the *Coreopsis*.

The area under the curve (AUC) values for the six distribution prediction models of *Coreopsis* plants, constructed using MaxEnt software. All the obtained scores were > 0.98 (Table 1), indicating that the high model has accurate and reliable simulation results. The MaxEnt model was employed to analyze the major environmental variables limiting the presence of *Coreopsis* species (Figure 2), as well as the specific values of each key environmental variable corresponding to suitable habitats (presence probability > 0.5) (see Table 1). Table 2, among the key environmental variables for *Coreopsis grandiflora* , isotherm (BIO3) and the mean temperature of the warmest season (BIO10) accounted for 81.6% of all variables. This indicates that temperature conditions are more influential than humidity in determining the geographic distribution of *C. grandiflora* . The mean temperature of the driest quarter (BIO9) at 23.1%, the mean temperature of the coldest quarter (BIO11) at 23%, and the precipitation coefficient of variation (BIO15) at 18.6%. This shows that both temperature and humidity conditions are equally important in shaping the geographic distribution of *Coreopsis drummondii* . Human activity (HA) is also a significant factor affecting the distribution of *Coreopsis* species. It has a contribution of 37.1% in *C. grandiflora* , 42.9% in *C. lanceolata* , 40.4% in *C. tinctoria* , and 42.1% in *C. verticillata* distribution.

**TABLE 1:** Number of effective records, the AUC value of *Coreopsis*, and the threshold for dividing suitable from unsuitable areas.

Species	Distribution records	AUC	MTSS
*C. drummondii*	12	0.9823	0.3148
*C. grandiflora*	249	0.9827	0.3339
*C. lanceolata*	211	0.9838	0.3235
*C. major*	5	0.9941	0.2086
*C. tinctoria*	249	0.9814	0.2944
*C. verticillata*	24	0.9953	0.2278

**TABLE 2:** The main environmental variables that determine the distribution of the *Coreopsis* (top 5).

Species	Variable	Percent contribution/%	Suitablity more than 50%
*C. drummondii*	BIO09	23.1	−9.13 ~ 12.38°C
	BIO11	23	−9.76 ~ 10.43°C
	BIO15	18.6	> 78.79
	BIO02	10.6	< 10.42°C
	BIO18	6.6	379.54 ~ 2973.73 mm
*C. grandiflora*	HA	37.1	45.49 ~ 167.89
	BIO18	28.7	379.54 ~ 637.79 mm
	BIO04	15.5	7.65 ~ 11.19°C
	BIO15	9.9	45.56 ~ 60.06, 94.23 ~ 142.44
	BIO09	3	−0.78 ~ 11.82°C
*C. lanceolata*	BIO18	37.9	379.54 ~ 614.31 mm
	HA	27.2	41.82 ~ 174.62
	BIO04	19.3	7.20 ~ 11.02°C
	BIO15	9.4	48.83 ~ 63.34, 85.51 ~ 138.69
	BIO06	1.2	−8.74 ~ 4.15°C
*C. major*	BIO03	44.5	< 25.95
	BIO10	37.1	> 25.75°C
	BIO18	7.5	> 426.50 mm
	BIO08	6.4	> 22.20°C
	HA	2	> 46.72
*C. tinctoria*	HA	40.4	44.27 ~ 198.49
	BIO18	23.2	356.06 ~ 849.08 mm
	BIO04	14.6	7.65 ~ 11.47°C
	BIO15	10.1	70.83 ~ 148.99
	BIO09	2.5	−2.27 ~ 11.64°C
*C. verticillata*	HA	42.1	47.94 ~ 159.94
	BIO18	35.4	379.54 ~ 637.79 mm
	BIO04	12.3	7.26 ~ 11.47°C
	BIO15	5.4	52.58<
	BIO03	1.9	22.47 ~ 30.96

### 3.2 Potential Distribution Predictions Under Current Climate Scenario

**FIGURE 3:**
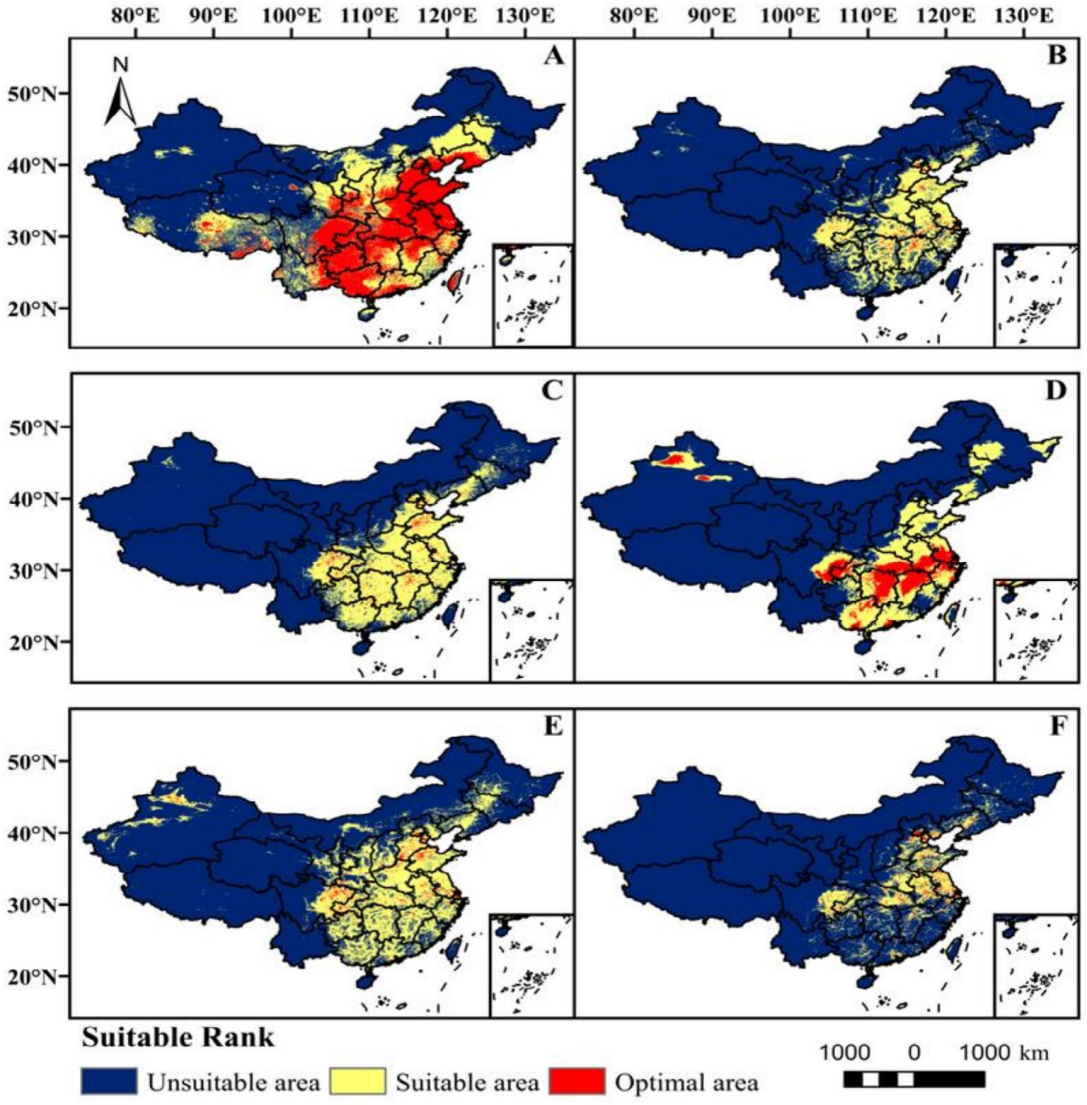
Potential distribution areas of six species of the Coreopsis under current climate scenarios. (A) *C. drummondii* ; (B) *C. grandiflor*; (C) *C. lanceolata* ; (D) *C. major* ; (E) *C. tinctoria* ; (F) *C. verticilla*.

Under the current climate scenario, the potential distribution areas for six *Coreopsis* species are mainly concentrated in the southern regions of China (Figure 3). Among these species, *Coreopsis drummondii* has the largest habitat suitability area, covering 4.138 × 10^6^ km^2^ (Table 3), and its suitable habitats are found in all provinces of China. Its optimal area is the largest among these six species, reaching 2.036 × 10^5^ km^2^ (Figure 3A). The next largest species is *Coreopsis tinctoria* , with a habitat suitability area of 2.436 × 10^5^ km^2^ (Table 3), and it is primarily distributed south of the Huanghe River. Furthermore, its optimal habitats are concentrated in the southeastern parts of Hebei, Shandong, Sichuan, Jiangsu, Anhui, and Zhejiang provinces (Figure 3E). *Coreopsis grandiflora* has a habitat suitability area of 2.082 × 10^5^ km^2^ (Table 3), and it is mainly distributed at the borders of Hubei, Hunan, Anhui, Jiangxi, Zhejiang, Jiangsu, Sichuan, and the Xinjiang Plateau (Figure 3D). *Coreopsis grandiflora* has a habitat suitability area of 1.638 × 10^5^ km^2^ (Table 3), which is mainly concentrated in the central‐southern region, with its optimal area being the smallest among the six species, at 6.76 × 10^4^ km^2^ (Figure 3B). *Coreopsis lanceolata* has a habitat suitability area of 2.141 × 10^5^ km^2^ (Table 3), and it is mainly distributed in central and southern China, and southern Liaoning. *Coreopsis verticillata* has the smallest habitat suitability area (9.53 × 10^4^ km^2^) (Table 3), which is mainly distributed in central China. Its optimal areas are primarily in the southwestern parts of Hebei, Henan, Zhejiang, and southwestern provinces (Figure 3F).

**TABLE 3:** The potential distribution area and extent of six species of the *Coreopsis* under three climate scenarios (SSP126, SSP245 and SSP585) from the current period (1970–2000) to the future period (2081–2100).

Species	Period	Climate scenarios	Suitable area	Optimal area	Range expansion	No change	Range contraction
*C. drummondii*	Current	—	210.20	203.60	—	—	—
		SSP126	158.47	201.17	17.24	357.73	70.45
	2041–2060	SSP245	340.94	309.77	230.81	426.21	1.97
		SSP585	220.98	280.16	110.59	398.22	29.95
		SSP126	245.66	229.37	84.81	401.39	26.79
	2081–2100	SSP245	355.24	281.96	220.17	418.93	9.25
		SSP585	355.73	457.55	380.41	426.87	1.31
*C. grandiflora*	Current	—	157.03	6.76	—	—	—
		SSP126	199.03	12.21	59.43	154.16	17.51
	2041–2060	SSP245	206.71	15.86	69.67	154.6	17.06
		SSP585	207.08	25.16	74.78	157.53	14.14
		SSP126	181.88	8.14	44.99	146.64	25.02
	2081–2100	SSP245	229.83	16.76	93.96	150.93	20.73
		SSP585	233.05	50.79	136.37	139.94	31.73
*C. lanceolata*	Current	—	204.75	9.38	—	—	—
		SSP126	210.23	23.26	33.42	207.11	18.36
	2041–2060	SSP245	232.92	24.49	52.5	211.43	14.04
		SSP585	251.79	20.31	67.45	207.95	17.52
		SSP126	252.63	15.62	64.02	209.37	16.1
	2081–2100	SSP245	269.98	23.43	91.91	201.84	23.63
		SSP585	258.51	98.97	151.7	199.7	25.78
*C. major*	Current	—	152.76	55.46	—	—	—
		SSP126	154.31	93.31	40.37	211.65	4.65
	2041–2060	SSP245	183.42	113.52	82.8	216.16	0.14
		SSP585	158.99	149.64	93.14	216.19	0.11
		SSP126	161.00	103.00	55.72	211.88	4.42
	2081–2100	SSP245	175.60	140.88	99.73	216.15	0
		SSP585	79.15	196.37	77.15	196.25	20.06
*C. tinctoria*	Current	—	231.76	11.83	—	—	—
		SSP126	304.47	38.65	106.05	238.65	12.49
	2041–2060	SSP245	286.13	63.39	109.68	239.59	11.55
		SSP585	343.34	51.14	32.72	164.98	86.16
		SSP126	312.55	37.07	111.29	241.36	9.78
	2081–2100	SSP245	311.24	58.23	137.64	226.69	24.45
		SSP585	315.13	142.14	232.46	216.21	34.93
*C. verticillata*	Current	—	86.90	8.40	—	—	—
		SSP126	86.16	13.37	18.26	82.34	16.3
	2041–2060	SSP245	87.94	13.91	23.01	78.89	19.75
		SSP585	111.01	23.22	42.72	90.79	7.85
		SSP126	84.06	10.45	22.83	71.77	26.87
	2081–2100	SSP245	105.97	19.56	36.46	89.23	9.41
		SSP585	151.20	79.11	129.95	96.21	2.43

### 3.3 Changes in the Distribution Range of *Coreopsis* Species Under Future Climate Change

**FIGURE 4:**
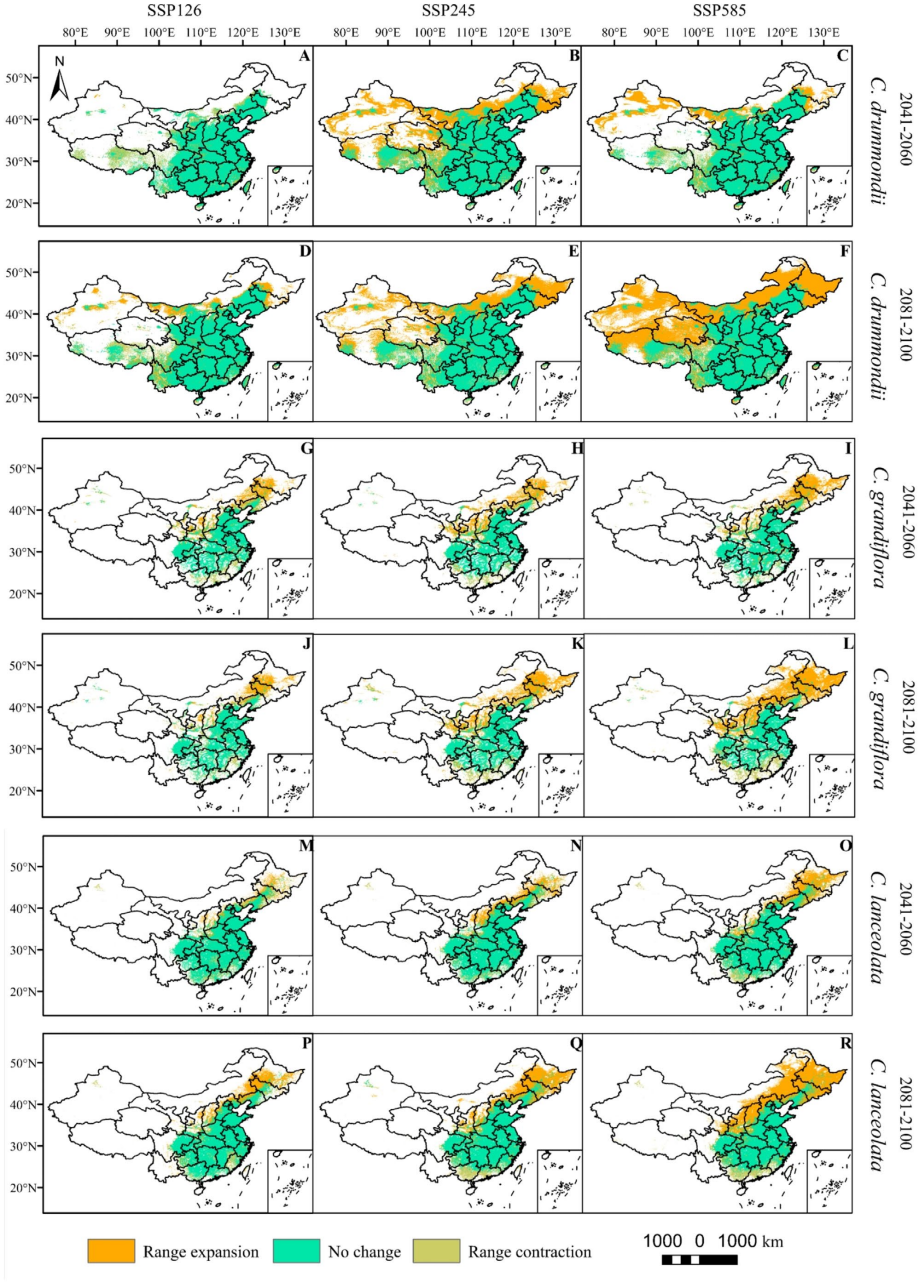
SSP126, SSP245, and SSP5853 scenarios show the range of *C. drummondii* , *C. grandiflora* , and *C. lanceolata* from the near current period (1970–2000) to the future period (2081–2100). (A–F) *C. drummondii* ; (G–L) *C. grandiflora* ; (M–R) C. *lanceolata* .

**FIGURE 5:**
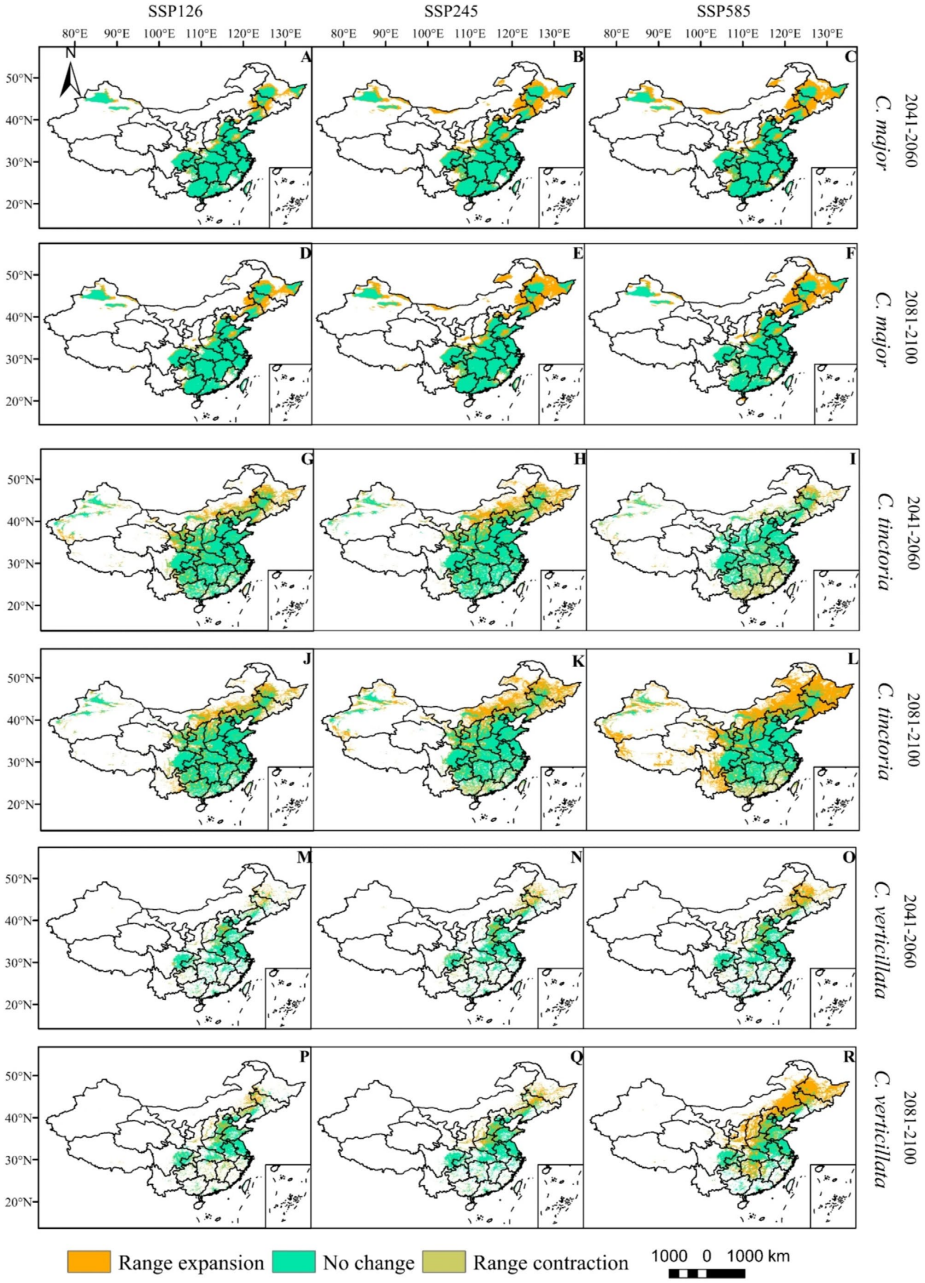
The range of *C. major* , *C. tinctoria* and *C. verticillata* under SSP126, SSP245, and SSP5853 scenarios from the near current period (1970–2000) to the future period (2081–2100). (A–F) *C. major* ; (G–L) *C. tinctoria* ; (M–R) *C. verticillata* .

The total potential distribution areas of *Coreopsis drummondii* and *Coreopsis tinctoria* will expand by the end of 2060 (Figures 3 and 4, Table 3). *Coreopsis drummondii* will have a nationwide distribution and will gradually expand (Figure 4A–C), while *Coreopsis tinctoria* will extend toward the northeast and northwest, primarily in high‐latitude regions (Figure 5G–I). Under the SSP245 and SSP126 climate scenarios, the total potential distribution areas of *Coreopsis grandiflora* and *Coreopsis lanceolata* will decrease, while under other climate scenarios, these areas will increase (Figure 4, Table 3). Their potential suitable regions will primarily expand toward higher latitudes and shrink at lower latitudes (Figure 4G,H,M–O). Under both the SSP245 and SSP126 climate scenarios, the potential suitable habitats of *Coreopsis verticillata* will shrink, with reductions mainly concentrated in the low‐latitude regions. Under the SSP585 climate scenario, the potential suitable habitat of *Coreopsis grandiflora* will expand, primarily extending toward higher latitudes (Figure 5G–I, Table 3).

The total area of suitable habitats for *Coreopsis drummondii* , *Coreopsis lanceolata* , and *Coreopsis grandiflora* will expand by 2100 (Figures 4 and 5, Table 3). The potential distribution area of *Coreopsis tinctoria* will mainly expand toward higher latitudes, particularly in the northeastern region, while the potential distribution in the Tibet Autonomous Region will also gradually expand (Figure 4D–F). *Coreopsis drummondii* and *Coreopsis grandiflora* will mainly expand toward higher latitudes (Figure 5D–FJ–L). Under the SSP‐585 and SSP‐245 climate scenarios, the potential suitable growth areas for *Coreopsis verticillata* and *Coreopsis grandiflora* are projected to decrease, while in other climate scenarios, these areas are expected to expand (Figure 4, Table 3). Under all three climate scenarios, the suitable growth areas for *Coreopsis verticillata* and *Coreopsis grandiflora* will extend toward higher latitudes. Under the SSP585 scenario and potential scenarios, *Coreopsis grandiflora* will expand toward the northeastern region, whereas in the SSP245 and SSP585 scenarios, the distribution areas in lower latitudes will shrink.

### 3.4 Migration of the Distribution Centroids of *Coreopsis* Species Under Different Climate Scenarios

**FIGURE 6:**
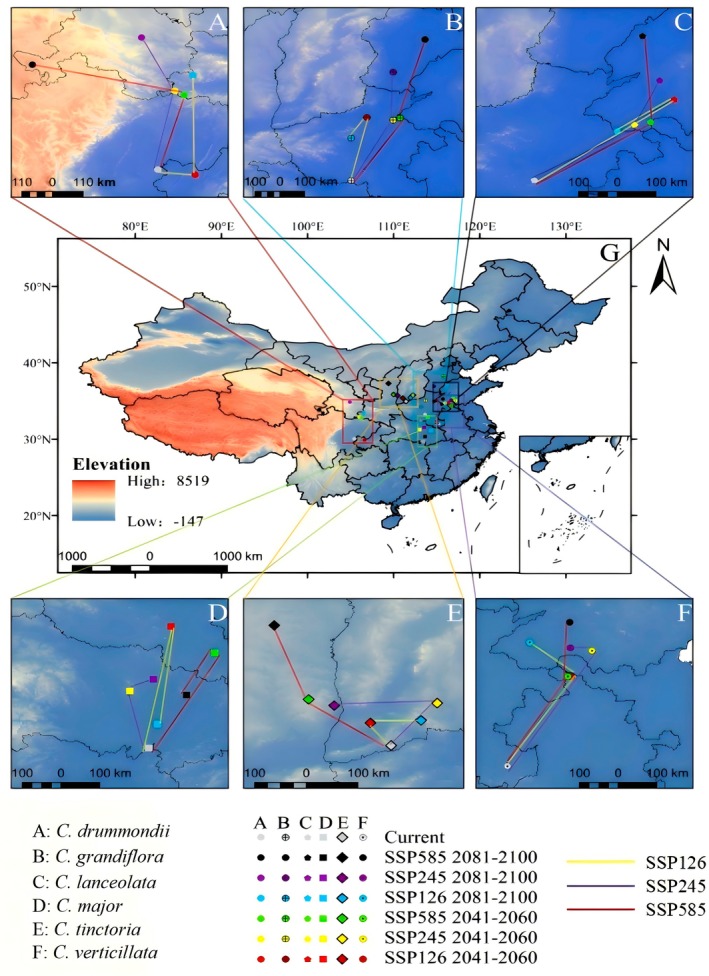
Centroid distribution of the *Coreopsis* under different climate change scenarios. (A): *C. drummondii* ; (B): *C. grandiflora* ; (C): *C. lanceolata* ; (D): *C. major* ; (E): *C. tinctoria* ; (F): *C. verticillata* ; (G): Distribution of 6 plant cores of the *Coreopsis* in China.

The results indicate that under the three future climate scenarios, the potential distribution centroids of six *Coreopsis* species will migrate to higher latitudes to varying extents (Figure 6). Under the current climate scenario, the distribution centroid of *Coreopsis drummondii* is located at the boundary of Sichuan and Guizhou provinces and it will shift toward the northwest in the future (Figure 6A). Currently, the distribution centroid of *Coreopsis grandiflora* is in the southern part of Henan province, and it will shift northward in the future. The northernmost centroid will move toward the southern part of Hebei province (Figure 6B). The distribution centroid of *Coreopsis lanceolata* is currently in the southern part of Henan province, and under all three future climate scenarios will migrate to higher latitudes (Figure 6C). The distribution centroid of *Coreopsis grandiflora* will generally shift northwest from 2041 to 2060, and toward the northeast from 2081 to 2100 (Figure 6D). The centroid of *Coreopsis tinctoria* is currently in the central region of Hubei province, and under all three future climate scenarios, it is expected to shift northward (Figure 6E). The centroid of *Coreopsis verticillata* is currently in the central region of Anhui province, and under all three future climate scenarios, it is expected to shift northward (Figure 6F).

### 3.5 Niche Comparison

**FIGURE 7:**
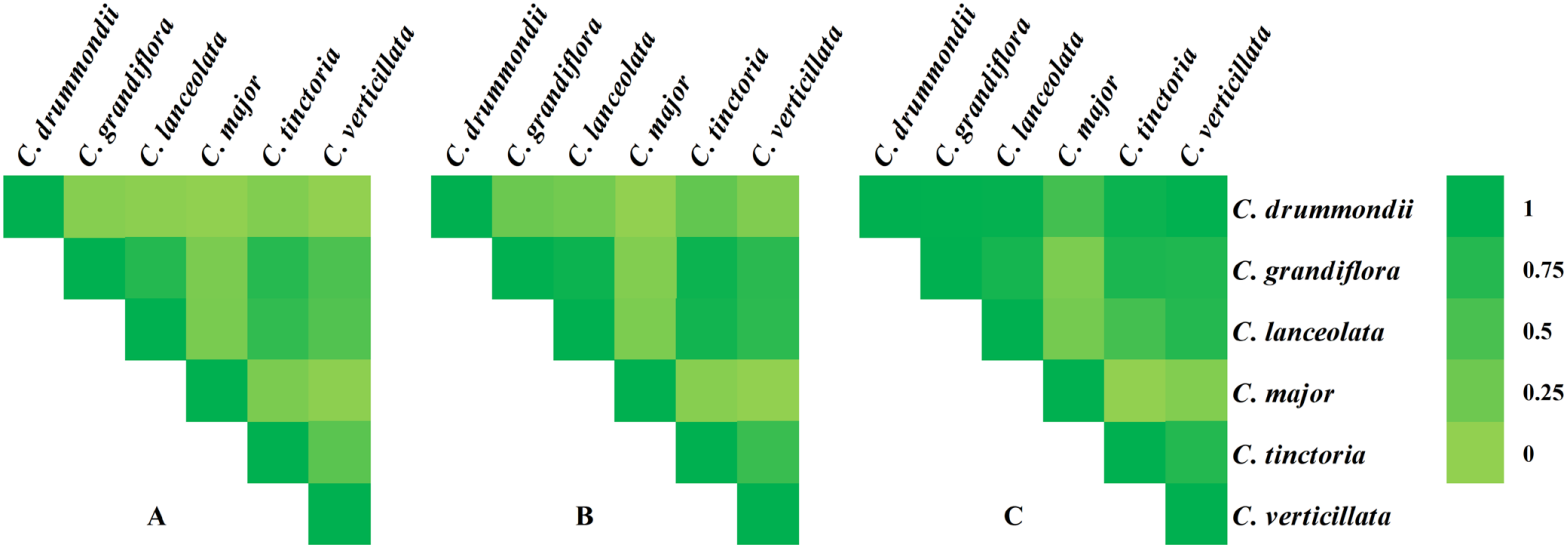
Interspecific niche overlap and range overlap of the *Coreopsis* in the current period. (A) Interspecific D value of the *Coreopsis*; (B) Interspecific I value of the *Coreopsis*; (C) Overlapping interspecific ranges of the *Coreopsis*.

The results showed that *Coreopsis grandiflora* has a high niche overlap followed by *Coreopsis lanceolata* (D: 0.85, I: 0.97) and *Coreopsis tinctoria* (D: 0.84, I: 0.97) (Figure 7). The niche overlap between *Coreopsis lanceolata* and *Coreopsis tinctoria* is also high (D: 0.79, I: 0.96). The niche overlap of *Coreopsis verticillata* with *Coreopsis grandiflora* (D: 0.67, I: 0.90) and *Coreopsis lanceolata* (D: 0.65, I: 0.89) is relatively high. The range overlap of *Coreopsis drummondii* with *Coreopsis grandiflora* , *Coreopsis lanceolata* , and *Coreopsis verticillata* is similar, each being 0.99. The range overlap between *Coreopsis grandiflora* and *Coreopsis lanceolata* (0.94), *Coreopsis tinctoria* (0.92), and *Coreopsis verticillata* (0.90) is relatively high.

## 4 Discussion

### 4.1 Influence of Dominant Environmental Factors on the Distribution of *Coreopsis* Species

Environmental factors play a crucial role in determining species' geographical distribution patterns and further influence the composition and dynamics of plant communities (Li and Park 2020; Ngarega et al. 2022). This study analyzed the influence of environmental factors on the spatial distribution of *Coreopsis* species under current climate conditions, revealing that both temperature and precipitation are significant factors shaping their distribution patterns. For *Coreopsis major* and *Coreopsis drummondii* , the influence of temperature conditions was more pronounced than that of precipitation. The primary environmental factors affecting *C. major* were isothermality (BIO3) and the mean temperature of the warmest quarter (BIO10), which together accounted for 81.6% of the contribution. Its wild populations are mainly distributed in Jiangxi Province, characterized by a subtropical monsoon climate with warm winters and summers. Higher isothermality may aid *C. major* in efficiently utilizing daytime temperatures for photosynthesis while reducing respiratory consumption during cooler nights, promoting nutrient accumulation and benefiting population reproduction (Yuan et al. 2024; Zhang et al. 2020). In contrast, the distribution of *C. drummondii* was jointly regulated by the mean temperature of the driest quarter (BIO09), the mean temperature of the coldest quarter (BIO11), and the precipitation seasonality (coefficient of variation, BIO15). This indicates that the distribution of *C. drummondii* is not primarily governed by common climatic indices like annual mean temperature or total annual precipitation, but rather by extreme seasonal climatic stresses encountered around its growing season and the stability of water availability.

The spatial distributions of the other four *Coreopsis* species showed greater responsiveness to precipitation conditions, particularly to precipitation of the warmest quarter (BIO18). Studies have shown that plants can utilize water conditions to regulate their growth, making water availability a key factor modulating plant development (Alinejad et al. 2020). These four species also exhibited some response to temperature seasonality (BIO04), as temperature is a major environmental factor influencing plant growth and development (Dawson et al. 2011). Furthermore, human activity (HA) significantly influenced the distribution patterns of these four species. *Coreopsis tinctoria* has been cultivated and utilized over a long period in Xinjiang due to its traditional medicinal values, such as anti‐inflammatory and antibacterial activities (Ye et al. 2025). Meanwhile, *C. grandiflora* , *C. lanceolata* , and *C. verticillata* were introduced to China as ornamental plants (Liu 2002; Ye 2007; Zhao 2017). Through artificial introduction, horticultural cultivation, and accompanying seed dispersal, not only has their suitable habitat range been expanded, but their potential invasive capacity has also been enhanced. Overall, despite belonging to the same genus, these species exhibit distinct response mechanisms to environmental factors. These differences likely stem from physiological and ecological traits formed during their respective evolutionary histories, thereby shaping different resource utilization strategies and geographical distribution patterns.

### 4.2 Differences in Potential Distribution Areas of Six *Coreopsis* Species Under Climate Change Scenarios

Predictions of the potential distribution areas for *Coreopsis* species under current climate conditions using the MaxEnt model revealed that they are primarily distributed in central and southern China (Figure 3), showing substantial overlap with their actual distribution records (Figure 1). Different species exhibited clear differentiation in their adaptability to future climates. Under the SSP585 scenario, the suitable distribution areas for *C. drummondii* and *C. tinctoria* during the period 2081–2100 showed significant growth, with the magnitude of range expansion far exceeding contraction, suggesting they may benefit from climate change. In contrast, *C. major* displayed a unique response pattern under the SSP585 scenario: its suitable habitat area drastically contracted (from the current 152.76 to 79.15 km^2^), while its optimal habitat area substantially increased (from 55.46 to 196.37 km^2^). This indicates that its future distribution may become highly concentrated and fragmented, with enhanced core habitat quality but compressed overall living space, facing a high risk of habitat loss. The suitable areas for *C. grandiflora* and *C. lanceolata* remained stable or showed slight increases across all scenarios, demonstrating strong climatic tolerance.

A key finding of this study is that for most species under climate change, the increase in “optimal habitat” was generally greater than or distinct from the change trend in “suitable area.” Alternatively, climate change may alter the intensity of human activities—including urban development, population density, cropland, pasture, roads, and railways (Gurney et al. 2022)—which could reduce the survival probability of these species and fragment their distributions. This suggests that although some species may maintain or even expand their distribution ranges, the geographical locations of their high‐quality core habitats (optimal habitats) may shift significantly or be redistributed. Such spatial restructuring of habitat quality may force species to migrate to track suitable climates, posing a significant challenge for plants with limited dispersal capacity. This could result in the actual inhabitable area being smaller than the potential suitable area predicted by the models.

Under future climate warming scenarios, the potential suitable habitats for most *Coreopsis* species showed a trend of expansion toward higher latitudes and altitudes, while suitable areas contracted at lower latitudes (Figures 5 and 6). Contraction in lower latitudes may be related to climatic variability leading to habitat degradation, ultimately causing regional extinction of the species (Parmesan and Hanley 2015). The northward expansion of suitable areas implies that regions previously free from invasion may face new ecological risks, and native species could be displaced due to a lack of competitive or adaptive mechanisms. Therefore, future monitoring and early warning efforts should be strengthened in potential invasion front areas, such as northeastern China, northwestern China, and the margins of the Qinghai‐Tibet Plateau.

### 4.3 Niche Overlap Suggests Potential Risk of Interspecific Competition

Climate change drives shifts in species' niches toward higher elevations or polar regions, potentially leading to niche overlap among closely related species (MacDonald et al. 2024). The results of this study indicate a high degree of niche and range overlap among *C. grandiflora* , *C. lanceolata* , and *C. tinctoria* . This suggests substantial similarity in resource utilization and ecological requirements among these congeneric species, which may trigger intense interspecific competition (Woo et al. 2024). In natural ecosystems, interspecific competition affects species distribution, population dynamics, and community structure (Barabás et al. 2016). When two or more species compete for the same limited resources, the less competitive species may be excluded or have its distribution restricted (Barabás et al. 2016; Ohba et al. 2022). Such competition could alter their population sizes and distribution patterns, further impacting the stability of the entire ecosystem. However, research on interspecific competition within the genus *Coreopsis* remains limited. Therefore, further investigation into the competitive strategies among different *Coreopsis* species is warranted to promote the conservation of wild resources.

## 5 Conclusion

In summary, temperature, precipitation, and human activities are key factors influencing the distribution of *Coreopsis* species. In the future, the potential distribution ranges of most *Coreopsis* species are projected to shift northward and toward higher altitudes, accompanied by a high degree of niche overlap among species. Future research should further investigate the interaction mechanisms between *Coreopsis* and native species and explore cultivation strategies to reduce invasion risks. Clarifying the environmental tolerance thresholds of different species will also provide a basis for accurately predicting distributional changes. Additionally, standardized management of ornamental *Coreopsis* species should be strengthened, and scientific cultivation protocols should be established. While this study provides insights into the distribution patterns of *Coreopsis* species, it has certain limitations. Subsequent research could refine the models and incorporate more factors to improve prediction accuracy. Against the backdrop of intensifying global change and human activities, enhanced research and monitoring of this genus are of significant importance for biodiversity conservation and ecosystem stability.

## Author Contributions

**Jinglin Jia:** investigation (equal), writing – original draft (lead), writing – review and editing (equal). **Junwei Ye:** software (equal). **Jianjun Zeng:** funding acquisition (lead), writing – review and editing (lead).

## Funding

This work was supported by National Natural Science Foundation of China, 32360264.

## Conflicts of Interest

The authors declare no conflicts of interest.

## Data Availability

All the required data are uploaded as Supporting Information.
